# Psychosocial outcome and health behaviour intent of breast cancer patients with *BRCA1/2* and *PALB2* pathogenic variants unselected by a *priori* risk

**DOI:** 10.1371/journal.pone.0263675

**Published:** 2022-02-15

**Authors:** Heamanthaa Padmanabhan, Nur Tiara Hassan, Siu-Wan Wong, Yong-Quan Lee, Joanna Lim, Siti Norhidayu Hasan, Cheng-Har Yip, Soo-Hwang Teo, Meow-Keong Thong, Nur Aishah Mohd Taib, Sook-Yee Yoon

**Affiliations:** 1 Genetic Counselling Unit, Cancer Research Malaysia, Subang Jaya, Selangor, Malaysia; 2 Core Laboratory Unit, Cancer Research Malaysia, Subang Jaya, Selangor, Malaysia; 3 Subang Jaya Medical Centre, Subang Jaya, Selangor, Malaysia; 4 Cancer Prevention and Population Science Unit, Cancer Research Malaysia, Subang Jaya, Selangor, Malaysia; 5 Department of Paediatrics, Genetic Medicine Unit, Faculty of Medicine, University Malaya Medical Centre, Kuala Lumpur, Malaysia; 6 Department of Surgery, Faculty of Medicine, University Malaya Medical Centre, Kuala Lumpur, Malaysia; University of Salerno, ITALY

## Abstract

There is an increasing number of cancer patients undertaking treatment-focused genetic testing despite not having a strong family history or high *a priori* risk of being carriers because of the decreasing cost of genetic testing and development of new therapies. There are limited studies on the psychosocial outcome of a positive result among breast cancer patients who are at low *a priori* risk, particularly in women of Asian descent. Breast cancer patients enrolled under the Malaysian Breast Cancer Genetic Study between October 2002 and February 2018 were tested for *BRCA1*, *BRCA2* and *PALB2* genes. All 104 carriers identified were invited by a research genetic counsellor for result disclosure. Of the 104 carriers, 64% (N = 66) had low *a priori* risk as determined by PENN II scores. Psychosocial, risk perception and health behaviour measures survey were conducted at baseline (pre-result disclosure), and at two to six weeks after result disclosure. At baseline, younger carriers with high *a priori* risk had higher Cancer Worry Scale scores than those with low *a priori* risk but all scores were within acceptable range. Around 75% and 55% of high *a priori* risk carriers as well as 80% and 67% of low *a priori* risk carriers had problems in the “living with cancer” and “children” psychosocial domains respectively. All carriers regardless of their *a priori* risk demonstrated an improved risk perception that also positively influenced their intent to undergo risk management procedures. This study has shown that with sufficient counselling and support, low *a priori risk* carriers are able to cope psychologically, have improved perceived risk and increased intent for positive health behaviour despite having less anticipation from a family history prior to knowing their germline carrier status.

## Background

In the past decade, the utility of germline genetic testing for cancer patients has expanded from risk management [1, 2] to treatment decision-making [3–6]. In addition, advancements in technology have led to the reduction in cost of testing, making it more affordable for the wider population [7, 8]. In the context of breast cancer, patients are traditionally referred from surgical and oncological clinics to genetics services for genetic counselling and testing. These patients are seen by genetic counsellors to ensure that they have sufficient information including benefits and limitations of genetic testing to make an informed decision about the test. These referrals are made based on guidelines such as the NCCN which is based on a set of criteria involving age of diagnosis, tumor pathological characteristics and family history of breast, ovarian, prostate and pancreatic cancers [9]. However, it has been shown that these criteria may miss 30% to 50% of carriers [10, 11]. This has impact in terms of treatment decision-making, as it was reported that up to 30% of patients who had universal genetic testing had modifications to their treatment based on their carrier status [10]. This proportion may increase further with the recent approval of Poly (adenosine diphosphate–ribose) polymerase (PARP) inhibitors as a treatment option for breast cancer patients who are germline *BRCA* carriers [12, 13]. In terms of cost, universal *BRCA* genetic testing was reported to be cost effective in upper and upper-middle income countries [14], and if the cost of testing is lowered further to a threshold of USD172 per test, it can be cost-effective in low-middle income countries as well [14]. As such, it is anticipated that there will be an increase in the demand for *BRCA* genetic testing for all breast cancer patients regardless of their *a priori risk*.

The increase in the use of genetic testing may enable more opportunities for risk management and treatment options but this could lead to potential harm to the patients’ and relatives’ psychological well-being. The psychosocial outcome and risk perception issues from *BRCA* genetic testing among breast cancer patients of high *a priori* risk have been well documented. These studies have shown that psychological outcomes such as distress, anxiety and depression among carriers did increased shortly after receiving results but reduced over time and was clinically insignificant in the intermediate and the long term [15, 16]. Previous reports have also shown that uptake of risk management strategies such as prophylactic surgery and increased screening depended on risk perception [17–20]. In some populations, it has been shown that women with no family history may be particularly vulnerable to adverse psychological impact arising from genetic testing [21]. As these studies are based on genetic testing offered to women who fulfilled guideline-based risk criteria, there is a knowledge gap of the psychosocial outcome and risk perception of breast cancer patients who do not fulfill risk-based testing criteria [21]. As genetic testing is increasingly offered to patients for both treatment selection and risk management purposes, it is important to understand the psychosocial outcome among individuals who may not have expected an inherited cancer risk.

The primary objective of this study was to determine factors that influenced cancer worry and risk perception of Asian breast cancer patients in relation to *a priori* risk, clinical and demographic characteristics. The secondary objective was to study the patients’ screening practices and health behaviour. These findings can provide information for genetic counsellors who provide psychosocial support to patients considering genetic testing.

## Methods

### Study population

Breast cancer patients with pathogenic variants in *BRCA1*, *BRCA2* or *PALB2* [22, 23] that were treated at University Malaya Medical Centre or Subang Jaya Medical Centre and participated in the Malaysian Breast Cancer Genetic (MyBrCa) Study [24] between October 2002 and February 2018 were included in this study. Carriers were approached by a researcher and invited to receive their genetic test results from a genetic counsellor and/or a clinical geneticist. Patients who agreed to receive their results were invited to complete questionnaires before the genetic counselling session (at baseline), and at follow-up (at two to six weeks post-result disclosure). Written informed consent was obtained from each patient who agreed to participate in this sub-study before baseline questionnaire was administered. This study was approved by the ethics committees of the University Malaya Medical Centre (UMMC 2017234884) and Subang Jaya Medical Centre (RSDH 201208.1).

### Determination of *a priori* risk

*A priori* risk of the participants was determined using PENN II [https://pennmodel2.pmacs.upenn.edu/penn2/] and they were categorised as high risk if they had PENN II scores ≥10 [25, 26].

### Psychosocial outcome measures

Psychosocial outcome of genetic testing and worry for recurrence were assessed using the Psychosocial Aspects of Hereditary Cancer Questionnaire (PAHC: 6 domains containing 26 questions, scored on a Likert scale of 1 to 4); and Cancer Worry Scale (CWS: 6 items, scored on a Likert scale of 1 to 4) respectively. Risk perception of the participants was evaluated using a 5-item Risk Perception Survey adapted from Evans et al., (1994) and Watson et al., (1999) [27, 28]. Health behaviour on screening practices and perceived usefulness of screening were evaluated using the 5-item Health Behaviour Survey adapted from Evans et al., (1994).

For the PAHC questionnaire, participants were considered to have a problem in a domain if one or more items within the domain scored ≥3 [29]. For Cancer Worry Scale, participants scoring ≥10 indicate concern for recurrence [30].

The Risk Perception Survey assesses participants’ understanding towards their own risk of inheriting a pathogenic variant, breast cancer and ovarian cancer risks compared to women of their age before and after knowing their genetic test result.

We evaluated the participants’ perception on the usefulness of breast and ovarian screenings (Mammogram, Magnetic Resonance Imaging, CA125 blood test and transvaginal ultrasound) using the Health Behaviour Survey. The survey also asked participants of their utilisation of breast and ovarian screenings within the past 6 months prior to result disclosure and their intention of undergoing these screenings within the next one-year post-result disclosure.

### Statistical methods

Participants’ characteristics and participants with high (≥10) and low (<10) CWS scores were compared using Chi-square test and Fisher’s exact test when the Chi-square test assumptions were not met for categorical variables and Welch’s t-test for continuous variables. We evaluated if there were changes in the proportions of individuals reporting problems in the six PAHC domains at baseline versus post-result disclosure using McNemar’s test. The statistical methods were performed using the R software version 3.6.3 and the descriptive analysis were conducted using Microsoft Excel 2013.

## Results

### Participants

Affected *BRCA1*, *BRCA2* and *PALB2* carriers who participated in the MyBrCa study were invited to receive their genetic test results. Of 104 carriers, majority were Chinese (N = 65, 63%) followed by Malays (N = 24, 23%), and Indians (N = 14, 14%). The average age of diagnosis of the carriers was 49.1 years. Majority had at least secondary education (80%), were married (87%) and had children (86%). Nearly one third (29%) reported family history of breast or ovarian cancer in first- or second- degree relatives (Table 1).

**Table 1:** Demographic characteristics of carriers.

Characteristics	All (N = 104)	Yes to result (N = 39)	No to result/ undecided (N = 46)	Deceased (N = 19)	*P*-value^a^
	N (%)	N (%)	N (%)	N (%)	
**Ethnicity**					*P*^1^ = 0.754; *P*^2^ = 0.013
Malay	24 (23.1)	7 (17.9)	9 (19.6)	8 (42.1)	
Chinese	65 (62.5)	28 (71.8)	30 (65.2)	7 (36.8)	
Indian	14 (13.5)	4 (10.3)	7 (15.2)	3 (15.8)	
Others	1 (1.0)	0 (0.0)	0 (0.0)	1 (5.3)	
**Age of diagnosis (years)**	49.1 ± 13.2	47.6 ± 10.8	53.1 ± 11.0	47.2 ± 11.7	*P*^1^ = 0.023; *P*^2^ = 0.258
**A *priori* risk**					*P*^1^ = 0.093; *P*^2^ = 0.624
High (PENN II ≥ 10)	38 (36.5)	21 (53.8)	9 (19.6)	8 (42.1)	
Low (PENN II < 10)	66 (63.5)	18 (46.2)	37 (80.4)	11 (57.9)	
**Education**					*P*^1^ = 0.014; *P*^2^ = 1.000
Primary or less	9 (20.0)	3 (13.6)	6 (30.0)	0 (0.0)	
Secondary​	22 (48.9)	8 (36.4)	12 (60.0)	2 (66.7)	
Tertiary^b^​	14 (31.1)	11 (50.0)	2 (10.0)	1 (33.3)	
Missing​	59​	17	26​	16	
**Income**					*P*^1^ = 0.290; *P*^2^ = 0.056
< RM5000	27 (60.0)	12 (54.5)	15 (75.0)	0 (0.0)	
≥ RM5000	18 (40.0)	10 (45.5)	5 (25.0)	3 (100.0)	
Missing	59​	17​	26	16	
**Marital status**					*P*^1^ = 0.032; *P*^2^ = 0.810
Married​	84 (86.6)​	30 (76.9)​	41 (95.3)​	14 (86.7)​	
Single​	10 (10.3)​	6 (15.4)​	2 (4.7)​	2 (13.3)​	
Divorced/ Separated​	3 (3.1)​	3 (7.7)​	0 (0.0)​	0 (0.0)​	
Missing​	7 ​	0 ​	3 ​	3 ​	
**Parity**					*P*^1^ = 0.131; *P*^2^ = 0.279​
Parous​	83 (85.6)	29 (80.6)	41 (93.2)	13 (76.5)	
Nulliparous​	14 (14.4)	7 (19.4)	3 (6.8)	4 (23.5)	
Missing​	7	3	2	2	
**Family history of breast or ovarian cancer** ^**c**^					*P*^1^< 0.001; *P*^2^ = 1.000
Yes	30 (28.8)	19 (48.7)	7 (15.2)	5 (21.1)	
None reported	74 (71.2)	20 (51.3)	39 (84.8)	14 (78.9)	
**Family history of other cancers** ^**d**^					*P*^1^ = 0.177; *P*^2^ = 0.849
Yes	32 (30.8)	9 (23.1)	17 (37.0)	5 (26.3)	
None reported	72 (69.2)	30 (76.9)	29 (63.0)	14 (73.7)	
**Tumor stage (AJCC6)**					*P*^1^ = 0.807; *P*^2^< 0.001
I	16 (23.5)	5 (22.7)	10 (30.3)	1 (7.7)	
II	26 (38.2)	10 (45.5)	15 (45.5)	1 (7.7)	
III	22 (32.4)	7 (31.8)	7 (21.2)	8 (61.5)	
IV	4 (5.9)	0 (0.0)	1 (3.0)	3 (23.1)	
Missing	36	17	13	6	

Abbreviations: N, Number of participants. *P*^1^ = P-value for “Yes to result” versus “No to result”; *P*^2^ = P-value for “Yes and No to result” versus “Deceased”.

^a^ P-values for Chi-square or Fisher’s exact test for categorical variables; and Welch’s t-tests for continuous variables.

^b^ Includes College (Diploma) and University.

^c^ First- and second- degree relatives.

^d^ Brain, colorectal, kidney, lung, nasopharyngeal, stomach, throat, thyroid, uterine cancers.

Of 104 carriers, 85 were alive and 19 deceased before the first contact. Deceased women were more likely to be diagnosed at later stages (84.6% vs 27.3%, *P* < 0.001; Table 1). Among the patients contacted, 39 (46%) came forward for genetic counselling and result disclosure, whereas 24 (28%) lost to contact and 22 (26%) declined to come in for counselling and result disclosure. These patients were diagnosed many years ago and the most common reason given for declining was that they have moved on from the diagnosis and would not like to have further conversations about it. Women who were younger (average age of diagnosis: 47.6 years vs 53.1 years, *P* = 0.023), with higher educational attainment (50% vs 10% with tertiary education, *P* = 0.014) and with family history of breast or ovarian cancer (49% vs 15%, *P* < 0.001) were more likely to come forward for genetic counselling. There was also a higher proportion of single women who came forward for genetic counselling (15.4% vs 4.7%, *P* = 0.032; Table 1).

### Psychosocial outcomes

**Fig 1:**
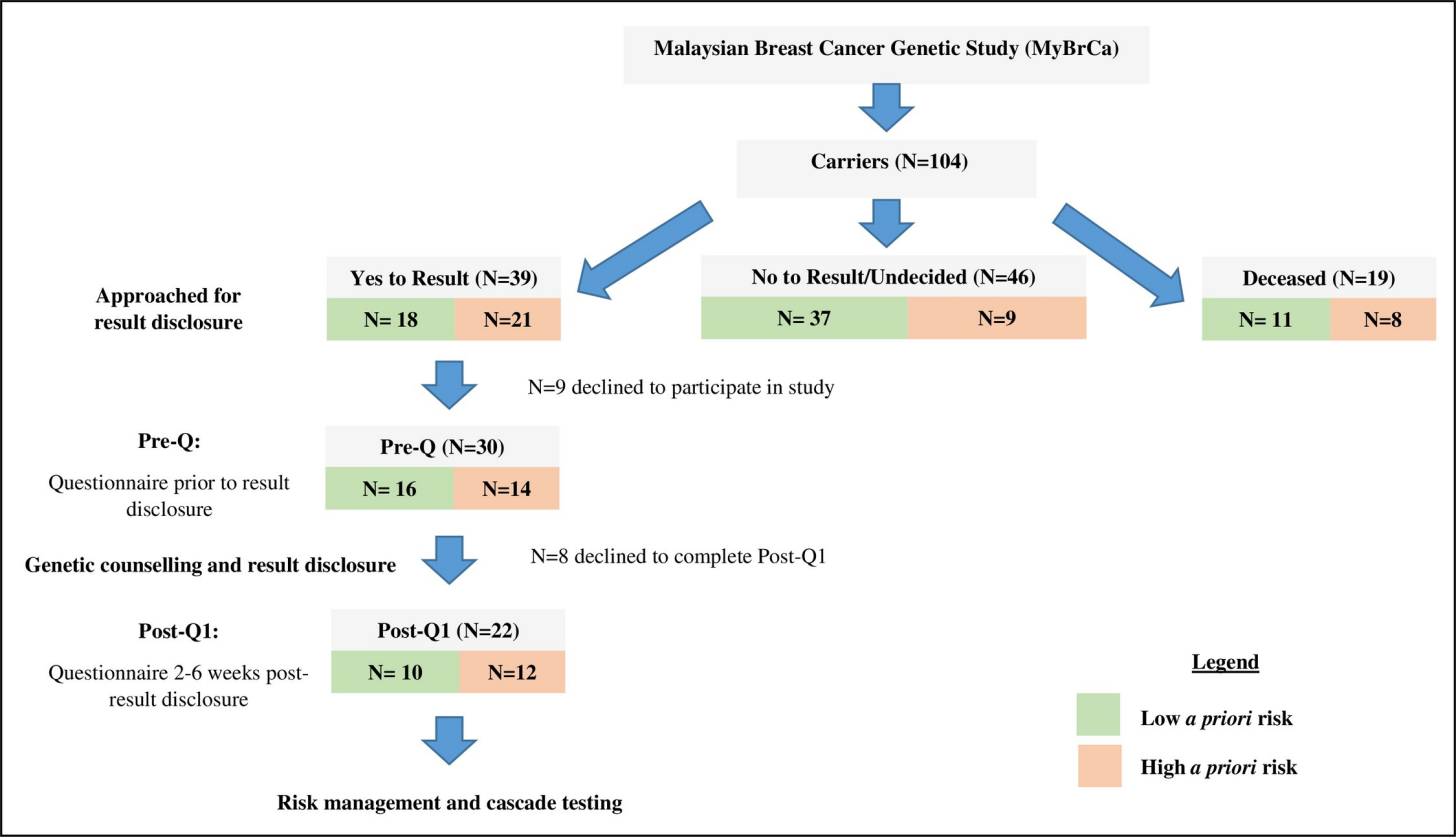
Study flow chart. Abbreviations: N, Number of participants.

Of the 39 patients counselled, 30 carriers agreed to participate in this study and completed a baseline questionnaire prior to result disclosure (Fig 1). We compared the demographic characteristics of individuals with high versus low CWS scores prior to attending genetic counselling (N = 30). Women who were younger at diagnosis (42.5 ± 9.8 vs 52.1 ± 11.5, *P* = 0.024) and have high *a priori* risk of being a carrier (8/11, 73% vs 6/19, 32%, *P* = 0.023; Table 2) had high CWS scores. However, there were no significant differences in the demographic characteristics between those with high and low CWS scores (Table 3) post-result disclosure.

**Table 2:** Cancer worry scores among carriers at baseline questionnaire.

Characteristics	All (N = 30)	High CWS scores (N = 11)	Low CWS scores (N = 19)	*P*-value^a^
	N (%)	N (%)	N (%)	
**Ethnicity**				0.824
Chinese​	22 (73.3)	9 (81.8)	13 (68.4)	
Malay	6 (20.0)	2 (18.2)	4 (21.1)	
Indian	2 (6.7)	0 (0.0)	2 (10.5)	
**Age of diagnosis (years)**	48.6 ± 11.7	42.5 ± 9.8	52.1 ± 11.5	0.024​
**A *priori* risk**				0.023​
High (PENN II ≥ 10)	14 (46.7)	8 (72.7)	6 (31.6)	
Low (PENN II < 10)	16 (53.3)	3 (27.3)	13 (68.4)	
**Education**				0.622​
Secondary or less	10 (58.8)	5 (71.4)	5 (50.0)	
Tertiary^b^	7 (41.2)	2 (28.6)	5 (50.0)	
Missing	13	4	9	
**Income**				1.000​
< RM5000	10 (58.8)	4 (57.1)	6 (60.0)	
≥ RM5000	7 (41.2)	3 (42.9)	4 (40.0)	
Missing	13	4	9	
**Marital status**				0.262
Married	22 (73.3)	8 (72.7)	14 (73.7)	
Single	5 (16.7)	3 (27.3)	2 (10.5)	
Divorced/ Separated	3 (10.0)	0 (0.0)	3 (15.8)	
**Parity**				0.372
Parous	23 (76.7)	7 (63.6)	16 (84.2)	
Nulliparous	7 (23.3)	4 (36.4)	3 (15.8)	
**Family history of breast or ovarian cancer** ^**c**^				1.000
Yes	12 (40.0)	4 (36.4)	8 (42.1)	
None reported	18 (60.0)	7 (63.6)	11 (57.9)	
**Family history of other cancers** ^**d**^				0.687
Yes	9 (30.0)	4 (36.4)	5 (26.3)	
None reported	21 (70.0)	7 (63.6)	14 (73.7)	
**Stage**				1.000
I	4 (21.1)	1 (14.3)	3 (25.0)	
II	8 (42.1)	3 (42.9)	5 (41.7)	
III	7 (36.8)	3 (42.9)	4 (33.3)	
Missing	11	4	7	

Note: High CWS score ≥ 10; Low CWS score < 10. Abbreviations: CWS, Cancer Worry Scale; N, Number of participants.

a *P*-values for Fisher’s exact test for categorical variables and Welch’s t-tests for continuous variables.

b Includes College (Diploma) and University.

c First- and second- degree relatives.

d Brain, colorectal, kidney, lung, NPC, stomach, throat, thyroid, uterine cancers.

**Table 3:** Cancer worry scores among carriers at post-questionnaire (N = 22).

Characteristics	All (N = 22)	High CWS (N = 8)	Low CWS (N = 14)	*P*-value^a^
	N (%)	N (%)	N (%)	
**Ethnicity**				0.602
Malay	4 (18.2)	2 (25.0)	2 (14.3)	
Chinese	18 (81.8)	6 (75.0)	12 (85.7)	
**Age of diagnosis (years)**	49.1 ± 11.9	44.1 ± 9.5	52.0 ± 12.6	0.114
**A *priori* risk**				0.183
High (PENN II ≥ 10)	12 (54.5)	6 (75.0)	6 (42.9)	
Low (PENN II < 10)	10 (45.5)	2 (25.0)	8 (57.1)	
**Education**				0.592
Secondary or less	8 (36.4)	3 (37.5)	5 (35.7)	
Tertiary^b^	5 (22.7)	3 (37.5)	2 (14.3)	
Missing	9 (40.9)	2 (25.0)	7 (50.0)	
**Income**				0.266
< RM5000	9 (40.9)	3 (37.5)	6 (42.9)	
≥ RM5000	4 (18.2)	3 (37.5)	1 (7.1)	
Missing	9 (40.9)	2 (25.0)	7 (50.0)	
**Marital status**				0.757
Married	17 (77.3)	6 (75.0)	11 (78.6)	
Single	4 (18.2)	2 (25.0)	2 (14.3)	
Divorced/ Separated	1 (4.5)	0 (0.0)	1 (7.1)	
**Parity**				1.000
Parous	17 (77.3)	6 (75.0)	11 (78.6)	
Nulliparous	5 (22.7)	2 (25.0)	3 (21.4)	
**Family history of breast or ovarian cancer** ^**c**^				0.675
Yes	10 (45.5)	3 (37.5)	7 (50.0)	
None reported	12 (54.5)	5 (62.5)	7 (50.0)	
**Family history of other cancers** ^**d**^				0.309
Yes	5 (22.7)	3 (37.5)	2 (14.3)	
None reported	17 (77.3)	5 (62.5)	12 (85.7)	
**Stage**				0.727
I	4 (18.2)	1 (12.5)	3 (21.4)	
II	3 (13.6)	0 (0.0)	3 (21.4)	
III	5 (22.7)	2 (25.0)	3 (21.4)	
Missing	10 (45.5)	5 (62.5)	5 (35.7)	

Note: High CWS score ≥ 10; Low CWS score < 10. Abbreviations: CWS, Cancer Worry Scale; N, Number of participants.

^a^*P*-values for Fisher’s exact test for categorical variables and Welch’s t-tests for continuous variables.

^b^ Includes College (Diploma) and University.

^c^ First- and second- degree relatives.

^d^ Brain, colorectal, kidney, lung, nasopharyngeal, stomach, throat, thyroid, uterine cancers.

**Fig 2:**
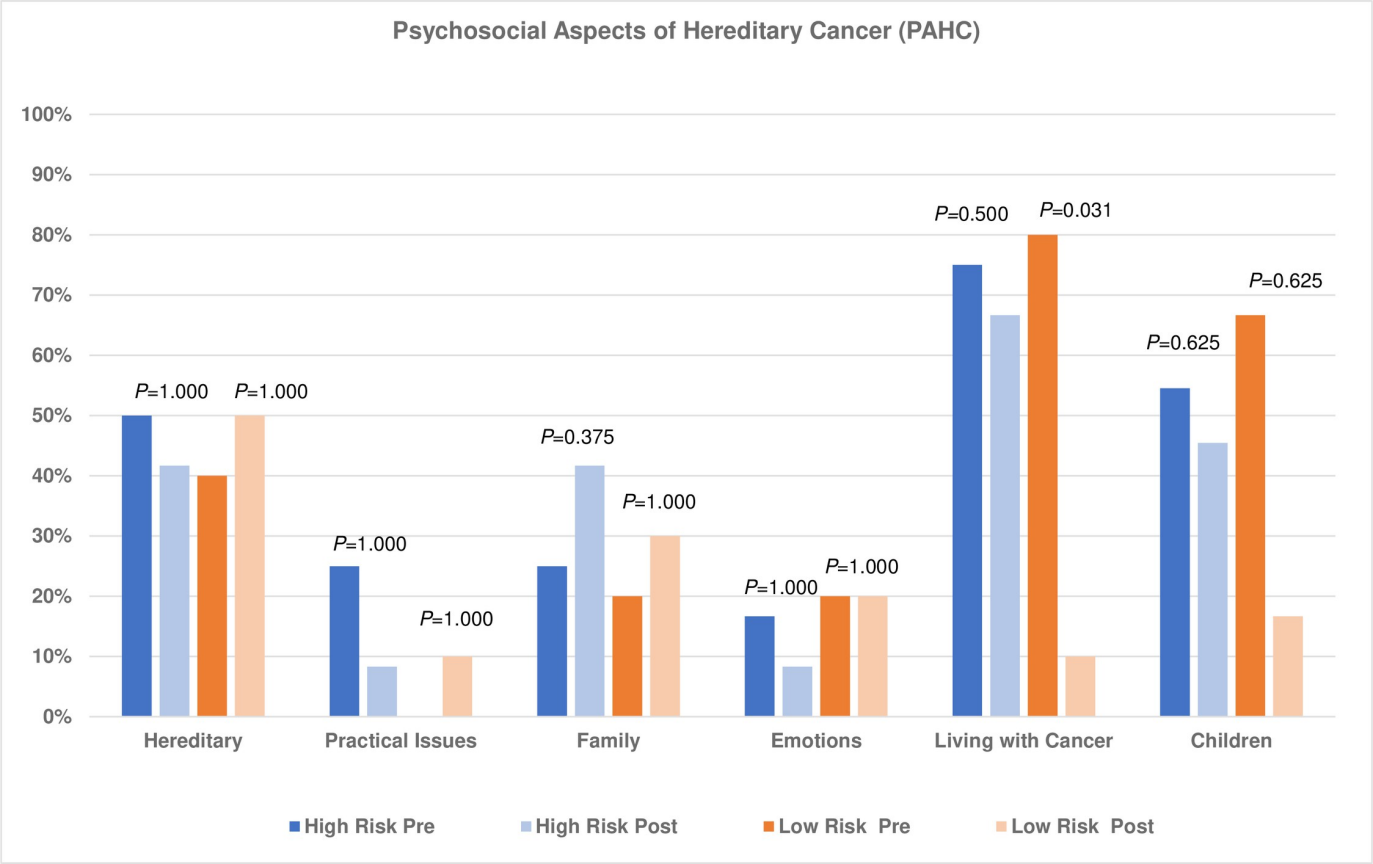
Psychosocial Aspects of Hereditary Cancer (PAHC) of 22 carriers pre- and post-result disclosure (two to six weeks after result disclosure). *P*-values for McNemar’s test comparing baseline and post-result disclosure for each domain for high and low *a priori* risk independently. High *a priori* risk carriers, N = 12; Low *a priori* risk carriers, N = 10.

We further explored the psychosocial outcomes among carriers according to their *a priori* risk. Of the 30 carriers who completed baseline, 22 (73%) proceeded to complete post-result questionnaire. Participants who did not complete the post-result questionnaire (N = 8) were either not contactable (50%) or reluctant to complete the questionnaire (50%). We observed that prior to result disclosure, both carriers with high (“high risk” HR) and low (“low risk” LR) *a priori* risk reported problems in the domains of living with cancer (HR: 75%, LR: 80%) and children (HR: 55%, LR: 67%). For both high and low *a priori* risk carriers, there was no significant difference observed in the majority of the domains except for living with cancer domain among the low *a priori* risk carriers (*P* = 0.031). There was an increase in problems in the family and social environment domain (25% to 41.7%) among the high *a priori* risk carriers (Fig 2). In contrast, for carriers with low *a priori* risk, there was a reduction in the domains of living with cancer (pre: 80%, post: 10%) and children (pre: 67%, post: 17%) after result disclosure. There was also marginal increase in problems reported post-disclosure in the hereditary (pre: 40%, post: 50%), practical issues (pre: 0%, post: 10%) and family and social environment (pre: 20%, post: 30%) domains among the low *a priori* risk carriers (Fig 2).

### Risk perception and health behaviour

**Fig 3:**
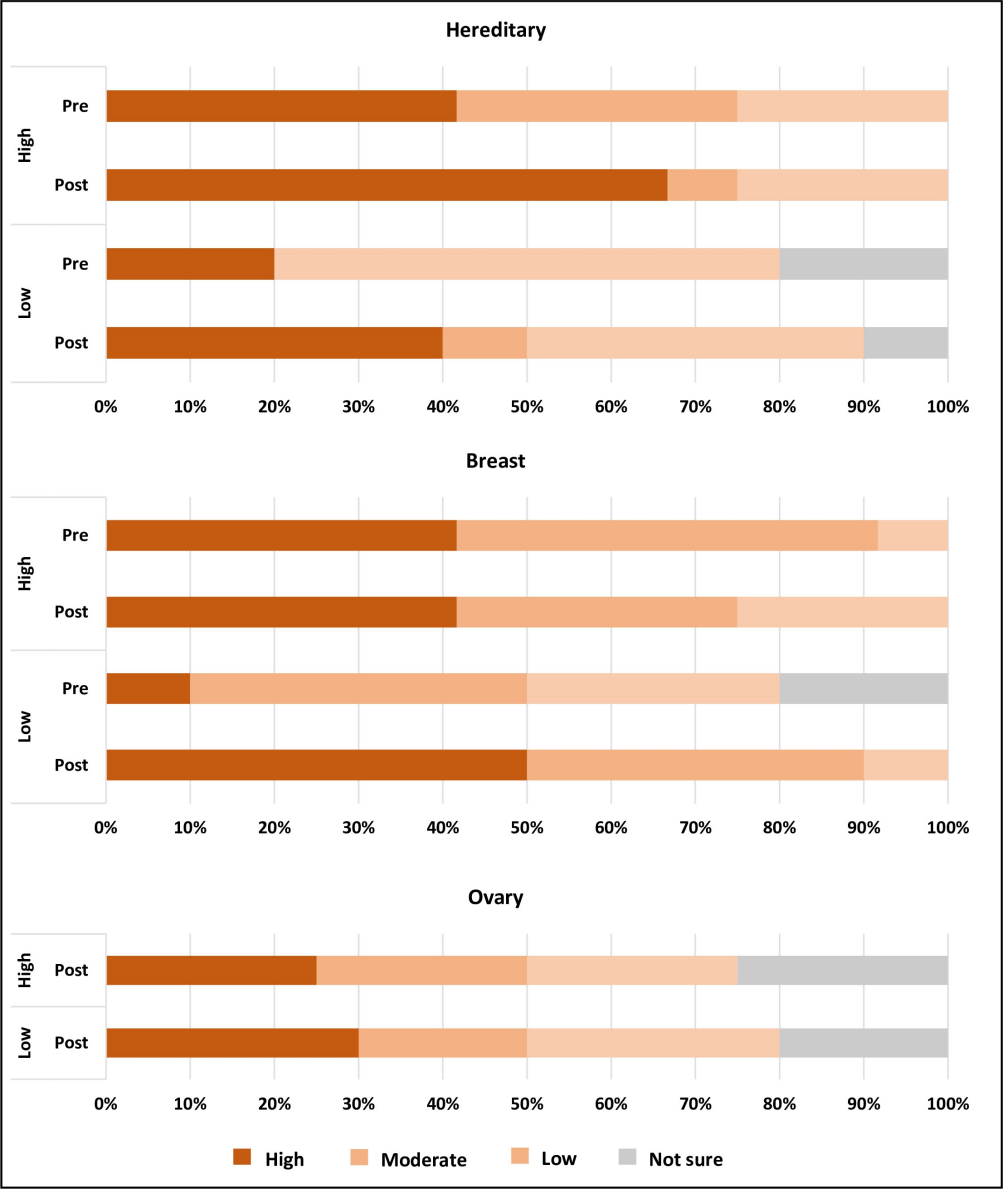
Risk perception of 22 carriers towards their own hereditary cancer, breast cancer and ovarian cancer risks. High *a priori* risk carriers, N = 12; Low *a priori* risk carriers, N = 10.

Of the 39 carriers counselled, 22 (56%) responded to the post-results questionnaire at follow-up (2 to 6 weeks post-result disclosure, Fig 1). We evaluated the carriers’ perception of their risk to breast and ovarian cancers prior to and after results disclosure. Among those with high *a priori* risk, the proportion of carriers who reported high perceived risk of hereditary cancers increased from 42% pre-result disclosure, to 67% post-result disclosure (Fig 3). Similarly, among those with low *a priori* risk, the proportion of carriers who reported high perceived risk of hereditary cancers increased after results disclosure, but these remained lower than for those with high *a priori* risk (pre: 20%, post: 40%; Fig 3). Carriers with high *a priori* risk reported high perceived risk of breast cancer compared to women of their age at both pre- and post-results disclosure (pre and post: 41.7%), whereas the proportion of carriers with low *a priori* risk that reported high perceived risk of breast cancer increased from 10% pre-result disclosure, to 50% after result disclosure (Fig 3). Notably, 25% of carriers with high *a priori* risk and 30% of carriers with low *a priori* risk regarded their risk of ovarian cancer as low (Fig 3) after result disclosure.

### Health behaviour survey (utilisation and intent)

**Fig 4:**
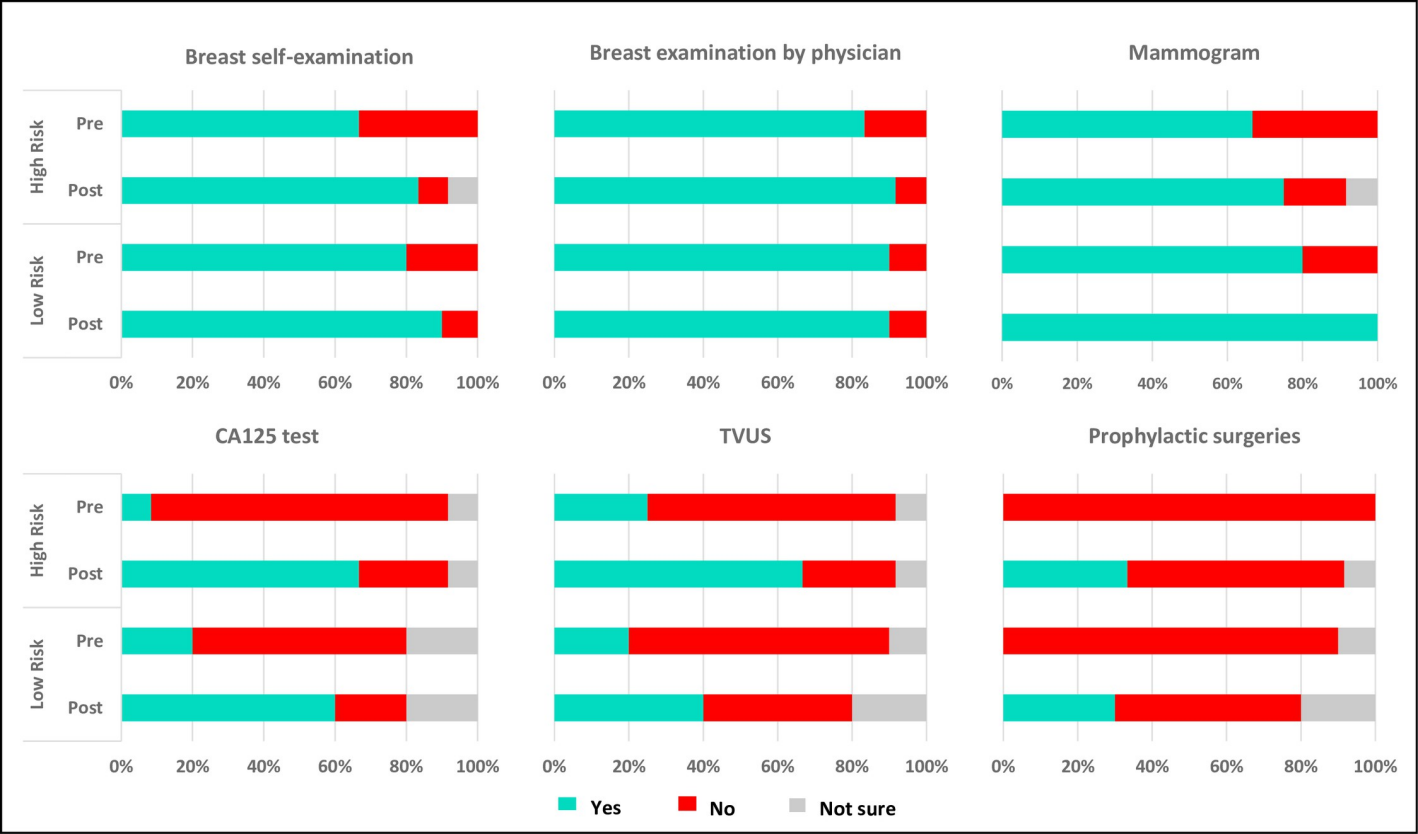
Utilisation (within the past 6 months prior to pre-questionnaire); intent (planning to carry out within the next 12 months after post-questionnaire) of screening and prophylactic surgery. High *a priori* risk carriers (N = 12) and Low *a priori* risk carriers (N = 10).

Of the 39 carriers who attended genetic counselling, 22 carriers (56%) responded to the health behaviour questionnaire prior to result disclosure and at follow-up (2 to 6 weeks post- result disclosure) (Fig 1). There was little difference between *a priori* high and low risk carriers in the uptake of self- or clinical breast examination, or of mammography screening prior to result disclosure and the intent remained high after result disclosure among all carriers regardless of *a priori* risk. In contrast, there was an increase in intent of ovarian cancer CA125 screening upon genetic counselling for carriers with high (pre: 7.1%, post: 66.7%) and low *a priori* risk (pre: 18.8%, post: 60%). Similar results were observed for transvaginal ultrasound screening (Fig 4). Notably, similar proportions of carriers with high and low *a priori* risk indicated intent to undergo prophylactic risk-reducing mastectomy (RRM) or risk-reducing bilateral salpingo-oophorectomy (RRBSO) surgeries after result disclosure (33.3% and 30% respectively, Fig 4).

## Discussion

In this study, the main areas of interest were in cancer worry, problems in psychosocial domains of “living with cancer” and “children related issues”, risk perception and health behaviour. Carriers who experienced higher CWS scores at baseline were younger and had high *a priori* risk, and majority of carriers regardless of their *a priori* risk reported problems in “living with cancer” and “children” related psychosocial domains prior to result disclosure. After result disclosure, women with both high and low *a priori* risk had a more accurate perception of their risk of hereditary cancers, breast and ovarian cancer especially among low *a priori* risk carriers. Despite having increased intent towards breast and ovarian cancer surveillance, only about one third of the carriers intended to undergo prophylactic RRM or RRBSO surgeries.

### Cancer worry

We found that high *a priori* risk carriers were more likely to have high CWS scores at baseline, however, after result disclosure no significant difference was observed between high and low *a priori* risk carriers. Interestingly, low *a priori* risk carriers did not show high CWS scores post-result disclosure, suggesting that individuals without high clinical risk were able to accept their carrier status with sufficient genetic counselling and psychosocial support. This is similar to a qualitative study conducted in an Ashkenazi Jewish population which showed that unaffected *BRCA* carriers with no strong family history viewed genetic testing positively. This was despite the challenges they faced during genetic counselling because genetic testing helped them plan for prevention and early detection strategies [31]. However, this is in contrast to another study conducted in Australia that reported women without strong family history were more distressed with their carrier status compared to those with family history and additional counselling sessions were recommended to this group of carriers to facilitate informed decision making in terms of risk management strategies and also to alleviate the psychosocial impact [21]. The low *a priori* risk carriers in our study, were provided with close attention during their genetic counselling sessions which may explain the acceptable CWS scores post result disclosure [30]. It may be appropriate to be offer genetic testing to those without high *a priori* risk provided that they have been well counselled.

### Psychosocial problem domain- living with cancer

Regardless of their *a priori* risk at baseline, living with cancer was the most prevalent problem among carriers and also after result disclosure for high *a priori* risk carriers. Notably, there is lack of long-term survivorship care in Malaysia in terms of psychosocial, physical and mental health support that are available to survivors [32]. They are often concerned about recurrence, risk of cancer in close family members, uncertainty of future and also face distress due to treatment side effects [32]. A recent study reported that breast cancer patients in Malaysia experience unmet needs in terms of psychological support and physical health needs as our Malaysian healthcare settings are not yet well established in providing psychosocial care for cancer patients [32, 33]. Therefore, non-governmental organisations and support groups are assuming the role of providing support financially, emotionally and physically [33]. Nevertheless, it is essential for genetic professionals and clinicians to pay attention to carriers regardless of their *a priori* risk in order to meet their psychological needs.

### Psychosocial problem domain- children related issues

The other psychosocial problem domain identified in this study was “children-related problems” This domain was also reported in Malaysian ovarian cancer patients [34] and in populations of European descent [29, 35]. In this study, carriers with children reported concern in informing them about their own genetic status and were worried if their children may develop cancer. This may be due to paternalistic relationship that is often seen between Asian parents and their children [36]. Therefore, patients with children may require additional counselling to cope with the communication of genetic information within the family.

### Risk perception and health behaviour

In this study, breast cancer patients’ perception on inheritance, and risk of breast and ovarian cancers improved after result disclosure. Patients also had increased awareness and intent of conducting breast and ovarian screening. Notably, carriers of low *a priori* risk underestimated their risk prior to result disclosure but this improved after counselling, demonstrating the need of genetic counselling to be conducted effectively to improve the accuracy of risk perception that eventually leads to positive health behaviour. Women with high risks of developing breast cancer, especially those with a family history of breast cancer, tended to correctly perceive their high-risk status, consistent with the literature [37, 38]. However, similarly in other populations, higher proportions of both high and low *a priori* risk carriers preferred screening and surveillance after knowing their carrier status rather than prophylactic surgeries [39]. There are limitations in the ovarian cancer screenings assessed in this study as their benefits are uncertain [11], hence, more time and attention should be poured into discussing prophylactic surgery for ovarian cancer among *BRCA* carriers who have completed child-bearing.

### Study limitations

There are several limitations in this study. First, the sample size of high and low risk carriers who participated in the questionnaire was small, which could limit the analysis. There was limited data in terms of the carriers’ adherence to surveillance and decision to undertake prophylactic surgeries, therefore the actual outcome of the patients’ health behaviour was not reported. Further qualitative analysis needs to be carried out to better understand the risk perception and health behaviour of the carriers. However, this study serves as an important provisional study to guide practice in universal genetic testing of breast cancer patients.

## Conclusion

Careful genetic counselling is warranted for patients with low *a priori* risk who require germline genetic testing for the purpose of treatment decision making. All carriers in this study regardless of their *a priori* risk demonstrated improved risk perception that also positively influenced their intent to undertake risk management procedures.

This study has showed that patients with low or high *a priori* risk had similar outcomes in terms of psychosocial outcomes, risk perception and health behaviour intent after result disclosure. Our study has shown that with sufficient counselling and support, low *a priori risk* carriers are able to cope psychologically, have improved perceived risk and increased intent for positive health behaviour despite having less anticipation from a family history prior to knowing their germline carrier status. In addition, the main issue reported by the carriers regardless of *a priori* risk were in the domains “living with cancer” and “concern for their children” prior to result disclosure. These issues will require additional attention in future genetic counselling sessions.

As such, genetic testing can be offered with careful genetic counselling to all breast cancer patients to maximise the utilisation of genetic testing for cancer treatment and prevention.

## Supporting information

S1 Table List of detected BRCA1, BRCA2 and PALB2 pathogenic variants of patients eligible in the study. Abbreviations: NA, Not Applicable.
(DOCX)

S1 Data (XLSX)

## List of abbreviations

*BRCA*: BReast CAncer gene
CA125: Cancer Antigen 125
CWS: Cancer Worry Scale
HR: High *a priori* risk
LR: Low *a priori* risk
MyBrCa: Malaysian Breast Cancer Genetic Study
PAHC: Psychosocial Aspects of Hereditary Cancer
*PALB2*: Partner and localizer of *BRCA2*
