# Supplementary material for: Psychosocial outcome and health behaviour intent of breast cancer patients with BRCA1/2 and PALB2 pathogenic variants unselected by a priori risk
Source: PLoS One. 2022 Feb 15;17(2):e0263675. doi: 10.1371/journal.pone.0263675 (PMC8846504; doi:10.1371/journal.pone.0263675)
Supplement: S1 Table — Abbreviations: NA, Not Applicable. (DOCX) [file pone.0263675.s001.docx]

| **Patient ID** | **Gene** | **Exon** | **Variant details (HGVS cDNA)** | **Variant details (HGVS Protein)** |
| --- | --- | --- | --- | --- |
| BRC1668 | BRCA1 | 12 | c.4327C>T | p.(Arg1443*) |
| BRC1699 | BRCA1 | 3 | c.115T>C | p.(Cys39Arg) |
| BRC1752 | BRCA1 | 10 | c.2070_2071delAA | p.(Arg691Thrfs*2) |
| BRC1784 | BRCA1 | 10 | c.3288_3289delAA | p.(Leu1098Serfs*4) |
| BRC1988 | BRCA1 | 10 | c.3607C>T | p.(Arg1203*) |
| BRC2227 | BRCA1 | 10 | c.1058G>A | p. (Trp353*) |
| BRC2229 | BRCA1 | 10 | c.2726dupA | p.(Asn909Lysfs*6) |
| BRC2231 | BRCA1 | Exon 21_22 del | NA | NA |
| BRC2246 | BRCA1 | 10 | c.2635G>T | p.(Glu879*) |
| [BRC2246A@BRC0967@BRC2371](mailto:BRC2246A@BRC0967@BRC2371) | BRCA1 | 10 | c.2635G>T | p.(Glu879*) |
| BRC2338 | BRCA1 | 19 | c.5251C>T | p.(Arg1751*) |
| SD0128 | BRCA1 | 10 | c.3869_3870delAA | p.(Lys1290Metfs*4) |
| SD0140 | BRCA1 | 10 | c.3008_3009delTT | p.(Phe1003*) |
| SD0264 | BRCA1 | 14 | c.4562_4567delinsGAGGAGCT | p.(Asn1521Argfs*28) |
| SD0406 | BRCA1 | 10 | c.4065_4068delTCAA | p.(Asn1355Lysfs*10) |
| SD0643 | BRCA1 | 4 | c.190T>C | p.(Cys64Arg) |
| SD0901 | BRCA1 | 10 | c.3424delG | p.(Ala1142Hisfs*13) |
| SD1188 | BRCA1 | NA | c.4987-2A>G | p.(Gly2508Ser) |
| SD1488 | BRCA1 | 2 | c.66dupA | p.(Glu23Argfs*18) |
| SD1910 | BRCA1 | 10 | c.2635G>T | p.(Glu879*) |
| SD1929 | BRCA1 | 4 | c.182G>A | p.(Cys61Tyr) |
| BRC0127 | BRCA2 | 15 | c.7467dupT | p.(Ile2490Tyrfs*7) |
| BRC0245 | BRCA2 | 13 | c.7007G>T | p.(Arg2336Leu) |
| BRC0382 | BRCA2 | 25 | c.9276T>G | p.(Tyr3092*) |
| BRC0406 | BRCA2 | 11 | c.2339C>G | p.(Ser780*) |
| [BRC0455@1199](mailto:BRC0455@1199) | BRCA2 | 11 | c.4872_4873delTG | p.(Glu1625Lysfs*13) |
| BRC0559 | BRCA2 | 11 | c.5047C>T | p.(Gln1683*) |
| BRC0970 | BRCA2 | 11 | c.6591_6592delTG | p.(Glu2198Asnfs*4) |
| BRC1155 | BRCA2 | 11 | c.5681dupA | p.(Tyr1894*) |
| BRC1266 | BRCA2 | 3 | c.262_263delCT | p.(Leu88Alafs*12) |
| BRC1317 | BRCA2 | 10 | c.823G>A | p.(Gly275Ser) |
| BRC1381 | BRCA2 | 11 | c.3596C>T | p.(Ala1199Val) |
| BRC1397 | BRCA2 | NA | c.631+1G>A | NA |
| BRC1506 | BRCA2 | 23 | c.9097delA | p.(Thr3033Leufs*29) |
| BRC1507 | BRCA2 | 23 | c.9097dupA | p.(Thr3033Asnfs*11) |
| BRC1564 | BRCA2 | 11 | c.3680_3681delTG | p.(Leu1227Glnfs*5) |
| BRC1576 | BRCA2 | 25 | c.9294C>A | p.(Tyr3098*) |
| BRC1589 | BRCA2 | 23 | c.9097dupA | p.(Thr3033Asnfs*11) |
| BRC1628 | BRCA2 | 11 | c.5727_5728insG | p.(Asn1910Glufs*2) |
| BRC1946 | BRCA2 | 10 | c.1565A>G | p.(Asp522Gly) |
| BRC1955 | BRCA2 | 15 | c.7516C>T | p.(Gln2506*) |
| BRC2107 | BRCA2 | 25 | c.9271_9274dupGTCT | p.(Tyr3092Cysfs*20) |
| BRC2123 | BRCA2 | 27 | c.9670delA | p.(Ile3224Tyrfs*25) |
| BRC2127 | BRCA2 | 11 | c.2521C>T | p.(Arg841Trp) |
| BRC2177 | BRCA2 | 11 | c.4914dupA | p.(Val1639Serfs*3) |
| BRC2259 | BRCA2 | 11 | c.2808_2811delACAA | p.(Ala938Profs*21) |
| BRC2269 | BRCA2 | 3 | c.262_263delCT | p.(Leu88Alafs*12) |
| BRC2288 | BRCA2 | 3 | c.262_263delCT | p.(Leu88Alafs*12) |
| BRC2326 | BRCA2 | 11 | c.6096dupT | p.(Ile2033Tyrfs*16) |
| BRC2327 | BRCA2 | NA | c.631+1G>A | NA |
| BRC2375 | BRCA2 | 11 | c.3136G>T | p.(Glu1046*) |
| BRC2386 | BRCA2 | 10 | c.809C>G | p.(Ser270*) |
| MyF066 | BRCA2 | 18 | c.8234_8237delTGAC | p.(Leu2745Glnfs*31) |
| MyF071 | BRCA2 | 11 | c.3195_3198delTAAT | p.(Asn1066Leufs*10) |
| MyF078 | BRCA2 | 11 | c.5851_5854delAGTT | p.(Ser1951Trpfs*11) |
| SD0163 | BRCA2 | 11 | c.2612C>A | p.(Ser871*) |
| SD0257 | BRCA2 | 11 | c.2286A>T | p.(Arg762Ser) |
| SD0320 | BRCA2 | 14 | c.7379_7382delACAA | p.(Asn2460Thrfs*8) |
| SD0401 | BRCA2 | 24 | c.9330dupT | p.(Glu3111*) |
| SD0589 | BRCA2 | 25 | c.9294C>A | p.(Tyr3098*) |
| SD0808 | BRCA2 | 11 | c.3847_3848delGT | p.(Val1283Lysfs*2) |
| SD1051 | BRCA2 | 11 | c.4467_4474delAATACTGAinsTGTTTTT | p.(Lys1489AsnfsTer15) |
| SD1121 | BRCA2 | 11 | c.4257delA | p.(Asp1420Ilefs*28) |
| SD1143 | BRCA2 | 14 | c.7379_7382delACAA | p.(Asn2460Thrfs*8) |
| SD1183 | BRCA2 | 10 | c.1368_1369delGA | p.(Lys457Alafs*4) |
| SD1189 | BRCA2 | 23 | c.8970G>A | p.(Trp2990*) |
| SD1240 | BRCA2 | 3 | c.2176delG | p.(Val726Phefs*4) |
| SD1349 | BRCA2 | 11 | c.5896dupC | p.(His1966Profs*2) |
| SD1357 | BRCA2 | 21 | c.8642delC | p.(Thr2881Lysfs*10) |
| SD1423 | BRCA2 | 11 | c.2588dupA | p.(Asn863Lysfs*18) |
| SD1431 | BRCA2 | 17 | c.7878G>A; | p.(Trp2626*) |
| SD1463 | BRCA2 | 11 | c.4829_4830delTG | p.(Val1610Glyfs*4) |
| SD1506 | BRCA2 | 11 | c.2808_2811delACAA | p.(Ala938Profs*21) |
| SD1684 | BRCA2 | 23 | c.8961_8964delGAGT | p.(Ser2988Phefs*12) |
| SD1782 | BRCA2 | 11 | c.3109C>T | p.(Gln1037*) |
| BRC0108 | PALB2 | 4 | c.839delA | p.(Asn280Thrfs*8) |
| BRC0261 | PALB2 | NA | c.3114-1G>A | NA |
| BRC0434 | PALB2 | 5 | c.1976_1977delTG | p.(Leu659Glnfs*3) |
| BRC0788 | PALB2 | 7 | c.2607delC | p.(Val870*) |
| BRC0945 | PALB2 | 4 | c.1037_1041delAAGAA | p.(Lys346Thrfs*13) |
| BRC0981 | PALB2 | 4 | c.1042C>T | p.(Gln348*) |
| BRC1055 | PALB2 | 1 | c.7G>T | p.(Glu3*) |
| BRC1069 | PALB2 | 11 | c.3166C>T | p.(Gln1056*) |
| BRC1126 | PALB2 | 4 | c.1050_1053delAACA | p.(Thr351Argfs*4) |
| BRC1133 | PALB2 | 13 | c.3543delT | p.(Phe1181Leufs*10) |
| BRC1184 | PALB2 | 2 | c.73A>T | p.(Lys25*) |
| BRC1868 | PALB2 | 4 | c.1059delA | p.(Lys353Asnfs*3) |
| BRC1869 | PALB2 | 4 | c.1059delA | p.(Lys353Asnfs*3) |
| BRC1945 | PALB2 | 4 | c.1037_1041delAAGAA | p.(Lys346Thrfs*13) |
| BRC2025 | PALB2 | 9 | c.2968G>T | p.(Glu990*) |
| BRC2130 | PALB2 | NA | c.211+1G>A | NA |
| BRC2221 | PALB2 | NA | c.211+1G>A | NA |
| BRC2306 | PALB2 | 13 | c.3543del | p.(Phe1181Leufs*10) |
| BRC2320 | PALB2 | 9 | c.2968G>T | p.(Glu990*) |
| BRC2434 | PALB2 | 9 | c.2968G>T | p.(Glu990*) |
| SD0221 | PALB2 | 8 | c.2760dupA | p.(Gln921Thrfs*7) |
| SD0311 | PALB2 | 1 | c.7G>T | p.(Glu3*) |
| SD0347 | PALB2 | 5 | c.2167_2168delAT | p.(Met723Valfs*21) |
| SD0483 | PALB2 | 4 | c.1050_1053delAACA | p.(Thr351Argfs*4) |
| SD0813 | PALB2 | NA | c.211+1G>A | NA |
| SD0996 | PALB2 | 9 | c.2968G>T | p.(Glu990*) |
| SD1054 | PALB2 | 5 | c.2167_2168delAT | p.(Met723Valfs*21) |
| SD1204 | PALB2 | 4 | c.426_428delGCTinsCC | p.(Lys142Asnfs*35) |
| SD1330 | PALB2 | 9 | c.2968G>T | p.(Glu990*) |

**S1_Table. List of Detected *BRCA1*, *BRCA2* and PALB2 Pathogenic Variants of patients eligible in the study.** Abbreviations: NA, Not Applicable.
